# Intra- and Inter-Host Assessment of *Bartonella* Diversity with Focus on Non-Hematophagous Bats and Associated Ectoparasites from Brazil

**DOI:** 10.3390/microorganisms8111822

**Published:** 2020-11-19

**Authors:** Priscila Ikeda, Jaire Marinho Torres, Lívia Perles, Elizabete Captivo Lourenço, Heitor Miraglia Herrera, Carina Elisei de Oliveira, Rosangela Zacarias Machado, Marcos Rogério André

**Affiliations:** 1 Laboratório de Imunoparasitologia, Departamento de Patologia, Reprodução e Saúde Única, Universidade Estadual “Júlio de Mesquita Filho”, Jaboticabal, São Paulo 14884-900, Brazil; prikeda@gmail.com (P.I.); liviaperles@hotmail.com (L.P.); rzacariasmachado@gmail.com (R.Z.M.); 2 Laboratório de Biologia Parasitária, Programa de Pós Graduação em Biotecnologia, Universidade Católica Dom Bosco, Campo Grande, Mato Grosso do Sul 79117-900, Brazil; jairemarinho@hotmail.com (J.M.T.); herrera@ucdb.br (H.M.H.); carinaelisei@yahoo.com.br (C.E.d.O.); 3 Laboratório de Ecologia de Mamíferos, Universidade do Estado do Rio de Janeiro, Rio de Janeiro 20550-013, Brazil; elizabete.c.lourenco@gmail.com

**Keywords:** genetic diversity, Chiroptera, *gltA*, *Bartonella*

## Abstract

The relationship among bats, ectoparasites and associated microorganisms is important to investigate how humans can become exposed to zoonotic agents. Even though the diversity of *Bartonella* spp. in bats and ectoparasites has been previously reported, the occurrence of *gltA* genotypes within hosts has not been assessed so far. We aimed to investigate the genetic diversity of *Bartonella* spp. in non-hematophagous bats and associated ectoparasites by assessing cloned *gltA Bartonella* genotypes in intra- and inter-hosts levels, as well as by using three additional molecular markers. Overall, 13.5% (18/133) bat blood samples, 17.18% bat flies (11/64) and 23.8% (5/21) Macronyssidae mite pools showed to be positive for *Bartonella* spp. Seventeen positive samples were submitted to *gltA-*cloning and three clones were sequenced for each sample. We also obtained 11, seven and three sequences for *nuoG*, *rpoB* and *ftsZ* genes, respectively. None were positive for the other target genes. We found at least two genotypes among the three *gltA*-cloned sequences from each sample, and 13 between all the 51 sequences. Among the *nuoG*, *rpoB* and *ftsZ* sequences we found eight, five and three genotypes, respectively. In the phylogenetic analysis, the sequences were positioned mainly in groups related to *Bartonella* identified in rodents, bats and bat flies. Herein, we showed the genetic diversity of *Bartonella* in bat’s blood and associated ectoparasites samples at both intra- and inter-host levels.

## 1. Introduction

The interest in emerging and reemerging diseases has increased since the late 20th century. It is estimated that 75% of the emerging infectious diseases are zoonoses, which are mainly transmitted by vectors. The main cause of zoonoses’ emergence is the human encroachment on natural habitats changing parasite-host relationships due to its fragmentation and their close contact with wildlife, domestic animals and humans [1].

*Bartonella* spp. are gram-negative, fastidious and facultative intracellular bacteria that can be transmitted by arthropod vectors. These bacteria primarily parasitize erythrocytes and endothelial cells, but can also colonize dendritic cells, macrophages and monocytes from different mammals and humans [2,3].

The order Chiroptera represents the second largest group of mammals in the world [4]. In Brazil, 9 families, 69 genera and 182 species of bats have been described so far in all morphoclimatic regions [5]. Due to their high mobility, wide distribution, social behavior, longevity [6] and unique immunological system [7], these animals are frequently associated with several emerging infectious diseases and pointed out as reservoir hosts and the source of infection for potential zoonotic agents [8,9]. Indeed, these animals have already been incriminated as possible reservoirs of a diversity of important bacteria, such as *Rickettsia* spp. [10], *Leptospira* spp. [11], hemotropic mycoplasma [12,13] and *Coxiella burnettii* [14]; protozoans, such as *Toxoplasma gondii* [15,16], *Leishmania* spp. [17,18,19,20,21] and *Trypanosoma* spp. [17,18,22,23,24,25,26,27]; and different fungi and viruses such as rabies [28]. Furthermore, it has been suggested that bats are the most probable ancestral hosts for eubartonellae and that their ability to fly may have contributed to making these mammals ideal hosts for the spread of infectious agents, such as those caused by arthropod-borne bartonellae [29].

Due to the high abundance of bat ectoparasites in Brazil, which comprises so far 96 Streblidae species [30], 26 Nycteribiidae species [31] and 32 species of mites (Acari) [32], it is important to achieve a better understanding of the relationship among bats, ectoparasites and associated microorganisms in order to understand the epizooetiology of such agents [33].

Genotypic diversity of *Bartonella* spp. in bats and associated ectoparasites has been widely reported [6,34,35,36,37,38,39]. Even though bartonellae genotypes have been detected in bats [12,40] and ectoparasites [41], in Brazil, the occurrence of multiple citrate synthase *gltA* genotypes in both vertebrate and invertebrate hosts infected by *Bartonella* spp. has not been assessed so far, since most studies in the literature have focused on the analysis of genetic diversity by targeting different *Bartonella* housekeeping genes, as previously proposed [42].

Therefore, this work aimed to investigate the genetic diversity of *Bartonella* spp. in non-hematophagous bats and associated ectoparasites from central-western Brazil. For this purpose, two different strategies were used: while the first one was based on the assessment of the genetic diversity of cloned *gltA Bartonella* genotypes at both intra- and inter-hosts levels, the second one investigated such diversity using a set of three genes.

## 2. Materials and Methods

All experimental procedures were approved by the “Instituto Chico Mendes de Biodiversidade” (ICMBio) (SISBIO 57450-1) and the Ethics Committee on Animal Use of the School of Agricultural and Veterinary Sciences, UNESP (CEUA FCAV/UNESP 010050/17).

Between June 2017 and March 2018, bats samplings were performed quarterly in two distinct places in the periurban region of Campo Grande city, Mato Grosso do Sul state, central-western Brazil, namely “Centro de Educação Ambiental Polonês” (CEA Pol.; 54°34′49.816″ W and 20°26′50.673″ S) and “Florestinha” (CEA Flor.; 54°33′43.352″ W and 20°24′11.614″ S). Lactating and/or pregnant females were released after field screening, as well as individuals who reached the maximum number of species collected per location as per license permits. The animals and their ectoparasites were identified using previously described identification keys for bats [43,44] and ectoparasites [45,46,47] (Table S1). The bats were then taken to the Laboratory of Parasitc Biology of the Catholic University Dom Bosco. Bats were euthanized using 10% ketamine and 1% acepromazine association at a 9:1 ratio and 1 mL/kg injected intramuscularly or intraperitoneally. From each bat specimen, 0.2–2 mL blood samples (according to the animal size) and spleen fragments were collected by intracardiac puncture and necropsy in EDTA and absolute ethanol (Merck^™^, Darmstadt, Germany) containing RNAse- and DNAse-free microtubes (Kasvi^®^, São José dos Pinhais, Brazil) and maintained at −80 °C and −20 °C, respectively, until their use in molecular assays. All samples were collected in laminar flow, using appropriate and sterilized instruments to avoid environmental contamination. A total of 418 biological samples represented by 135 spleen tissues, 133 whole blood and 150 ectoparasites (comprising 64 bat flies, 67 mite pools, and 19 ticks) were collected from 135 individuals and used in the present study to investigate the genetic diversity of *Bartonella* spp. The small body size of one animal and the difficulty of getting enough samples from another one (represented by specimens of *Myotis nigricans* and *Artibeus lituratus*, respectively) precluded DNA extraction from those blood samples.

For DNA extraction, 64 bat flies and 19 ticks were individually processed. On the other hand, Spinturnicidae (specimens belonging to the same species) and Macronyssidae (specimens belonging to the same genus) mites collected from the same individual were pooled in 46 and 21 samples, respectively. Each pool sample contained up to 50 mites. All ectoparasites and spleen samples were purified using Illustra Tissue and Cells Genomic Prep Mini Spin Kit (GE Healthcare™, Chicago, IL, USA), whereas bat blood samples were purified using the lllustra Blood Mini Spin Kit (GE Healthcare™, Chicago, IL, USA), following the manufacturer’s recommendations. DNA samples were evaluated by optical spectrophotometry (Nanodrop, Thermo Fisher Scientific™, Waltham, MA, USA) to assess the concentration (µg/µL) and 260/280—260/230 ratios.

The DNA samples were firstly tested by conventional (c) PCR assays based on endogenous genes to verify the absence of PCR inhibitors. In this sense, for the DNA samples obtained from blood and spleen tissue, the cPCR protocol based on the mammals-glyceraldehyde 3-phosphate dehydrogenase (*gapdh*) gene was performed as previously described [48]. For the ectoparasites collected from the animals, two different cPCR protocols were used. While a PCR protocol based on cytochrome c oxidase subunit I (*cox-1*) gene [49] was used for DNA samples obtained from bat flies and mites, a protocol based on the 16*S* rRNA gene [50] was used for DNA samples obtained from tick larvae. All the obtained 16S rRNA tick larvae amplicons were submitted to dideoxynucleotide chain termination [51] sequencing to check the molecular identity by nBLAST analysis.

The positive samples for the endogenous genes protocols were then submitted to a screening quantitative (q) PCR for *Bartonella* spp. based on the NADH dehydrogenase gamma subunit (*nuoG*) gene, as previously described [52]. All positive samples on the qPCR assay were tested by cPCR assays based on the following genes: *nuoG* [53], riboflavin synthase (*ribC*) [54], citrate synthase (*gltA*) [55], β-subunit of RNA polymerase (*rpoB*) [56], hemin binding protein (*pap-31*) [57], heat-shock chaperonin protein (*groEL*) [56,58], cell division protein (*fstZ*) [56], 16S rRNA [56] and intergenic region 16S–23S rRNA (ITS) [59], following the protocols previously described.

Products amplified in the cPCR assays were submitted to horizontal electrophoresis on ethidium bromide stained-agarose gels (1.0%) at 100 V/150 mA for 50 min.

*Bartonella-gltA* amplicons were submitted to the cloning protocol using the vector pGEM-T Easy (Promega™, Madison, WI, USA) following the manufacturer’s recommendations. The ligation products were inserted into competent *Escherichia coli* One Shot Match 1T1R Chemically Competent Cells (Invitrogen™, Carlsbad, CA, USA - Cat # C8620-03) (109–1010 cfu/ng of DNA) by thermal shock method. After seeding on a solid Luria Bertani (LB) medium prepared with 100 μg/mL ampicillin, 40 μL of X-gal (5-bromo-4-chloro-3-indolyl-β- D-galactoside) (Sigma-Aldrich™, St. Louis, MO, USA) and 20 μL IPTG (isopropylthio-β-galactoside) (Sigma-Aldrich™, St. Louis, MO, USA), the plates were incubated at 37 °C overnight. Three individual white colonies of bacteria were chosen for each sample and transferred into tubes containing LB liquid medium and 100 μg/mL ampicillin. After incubation at 37 °C for up to 24 h, plasmid DNA was extracted from the transforming clones by the alkaline lysis method [60].

The obtained amplicons were purified using the Silica Bead DNA gel extraction kit (Thermo Fisher Scientific™, Waltham, MA, USA) and submitted to sequencing by dideoxynucleotide chain termination [51], performed in ABI PRISM 3700 DNA Analyzer sequencer (Applied Biosystems™, Foster City, CA, USA) at the “Centro de Recursos Biológicos e Biologia Genômica (CREBIO-FCAV—UNESP)”. All the sequences obtained were evaluated and a consensus sequence was generated using the Phred-Phrap software version 23 (Genome Sciences Department, University of Washington, Seattle, WA, USA) [61,62]. Then, all obtained consensus sequences were compared with sequences previously deposited in the GenBank (National Center for Biotechnology Information, U.S. National Library of Medicine, Bethesda, MD, USA) database by nBLAST (National Center for Biotechnology Information, U.S. National Library of Medicine, Bethesda, MD, USA) [63].

The cloned sequences were aligned to evaluate the nucleotide polymorphism within each bat blood/spleen and ectoparasite sample and among all the cloned samples. The number of genotypes (h), genotype diversity (Hd) and nucleotide diversity (π) were determined using the DnaSP 5 program, version 5.10.01 (Universitat de Barcelona, Barcelona, Espanha) [64]. The genotype networks were plotted using PopART (New Zealand) (http://popart.otago.ac.nz) with TCS networks [65]. Additionally, for the *gltA* gene, a genealogy network was created using the software Splitstree v4.11.3 (Eberhard Karls Universität Tübingen, Tübingen, Germany) [66] using Neighbor-net method and bootstrap of 1000 replicates.

Phylogenetic inferences were performed for all four molecular markers from which sequences were obtained. The sequences were aligned with other sequences from the GenBank database through BioEdit [67]. The best evolutionary model was chosen by the jModelTest2 program (version 2.1.6) [68], under the Bayesian Information Criterion (BIC) [69]. The Bayesian inference (BI) analysis was performed with MrBayes 3.1.2 [70] using the CIPRES Science Gateway on XSEDE (The Extreme Science and Engineering Discovery Environment, USA) [71]. The phylogenetic tree edition and rooting (outgroup) were performed using the Treegraph 2.0 beta software [72]. The evolutionary models used for Bayesian Inferences were TVM, TIM2ef, TIM1 and TrN for *gltA*, *nuoG*, *ftsZ*, and *rpoB* genes respectively. All were settled with proportion of invariable sites and gamma rates.

The sequences were deposited in the GenBank database under accession numbers MT874166-MT874216 for *gltA* sequences, MT874217-MT874227 for *nuoG* sequences, MT874228-MT874234 for *rpoB* sequences and MT831908-MT831910 for *ftsZ* sequences.

## 3. Results

### 3.1. PCR Assays and Sequencing

All DNA samples were positive in the cPCR assay based on the endogenous genes (*gapdh* for the blood and spleen samples, 16S rRNA to the ticks samples, and *cox-1* to mite and fly samples). In qPCR assays for *Bartonella* spp. based on the *nuoG* gene, 34/418 (8.1%) samples showed positive results (efficiency ranging from 100.5% to 104.7%; slope from −3.311 to −3215; R^2^ from 0.969 to 0.997; *y*-intercept from 35.955 to 37.425). Overall, 18/34 (52.9%) were originated from bat blood samples and 16/34 (47.1%) were from ectoparasites samples. The bat blood positive samples [13.53% (18/133)] were represented by *Platyrrhinus lineatus* n = 12/23; *Artibeus planirostris* n = 3/33; *Artibeus lituratus* n = 2/37; and *Carollia perspicillata* n = 1/5. The positive bat flies [17.18% (11/64)] belonged to the following species: *Trichobius dugesii* complex n = 6/10; *Trichobius costalimai* n = 4/29; *Megistopoda aranea* n = 1/18*. Bartonella*-positive Macronyssidae pool mites [23.8% (5/21)] were represented by *Steatonyssus* spp. N = 5/21. One *P. lineatus* (#79) and one *C. perspicillata* (#42) each had the blood sample and associated ectoparasites (*T. dugesii* complex bat flies—one from animal #79 and two from the animal #42) positive in qPCR assay for *Bartonella* spp. No spleen samples, individual ticks and Spinturnicidae pool mites were positive for *Bartonella* spp. All the 34 positive samples were tested by cPCR to *Bartonella* spp. based on *gltA, nuoG, rpoB* and *ftsZ* genes. Due to the low volume of eluted DNA obtained in the nucleic acids extraction procedure from ectoparasites, they were not submitted to the cPCR protocols for *ribC*, *groEL*, *pap-31* and 16S rRNA genes and ITS region.

Even though 23 samples were positive in the cPCR assay based on *gltA* gene, 17 (14 from bats and 3 from flies) were submitted to cloning due to the quality of the amplicons observed in agarose gel electrophoresis. For this specific gene, three clones were sequenced from each sample, totaling 51 sequences that were used to determine the genotypic diversity among the different species of bats and flies studied as well as within each individual host. Similarly, although 13 and 15 samples were positive in the cPCR assays for the *rpoB* and *ftsZ* genes, seven (5 from bats and 2 from flies) and three sequences (2 from bats and 1 from flies) were obtained from each gene, respectively. All the samples submitted to the *nuoG* cPCR protocol were positive, except for the DNA samples from mites (Macronyssidae). Only those samples that did not show positive results for any other gene but yielded *nuoG* amplicons were submitted to sequencing, totaling 11 sequences (3 from bats and 8 from flies). None of the 18 *Bartonella nuoG* qPCR-positive bat blood samples were positive in the cPCR protocols based *ribC*, *groEL*, *pap-31* and 16S rRNA genes and ITS region (Table S2).

### 3.2. Sequences Homology Analysis

In the nBLAST analysis, three out of 51 *gltA* cloned showed 88.20%–88.55% identity (100% of query coverage) with *Bartonella vinsonii* strain NCTC12905 (GenBank Access Number LR134529). The 48 remaining *gltA* cloned sequences showed 91.29%–100% identity (query coverage value of 90%–100%) to *Bartonella* spp. sequences previously detected in bats and their ectoparasites sampled in Costa Rica (17/51 sequences showed identity to KJ816676—*Myotis keaysi*; 14/51 similar to MH234380—*Glossophaga soricina*; 5/51 were similar to KJ816682—*Artibeus jamaicensis*–*Aspidoptera phyllostomatis*; 3/51 sequences similar to MH234319—*A. lituratus*; 3/51 similar to MH234346—*C. perspicillata*; and 1/51 were similar to MH234347—*C. perspicillata*; 1/51 to MH234333—*Artibeus glaucus watsoni*; 1/51 to MH234322—*A. lituratus*; 1/51 to KJ816687—*A. lituratus–Paratrichobius longicrus*; 1/51 to KJ816666—*Anoura geoffroyi*; and 1/51 to KJ816673—*Sturnira lilium–Aspidoptera delatorrei*) (Table S3).

Regarding the *nuoG* gene, nine sequences showed 92.7%–98.98% identity (query coverage of 86%–100%) to *Bartonella* spp. previously detected in blood from vampire bats *Desmodus rotundus* (GenBank Access Number: MK314980/MK314982) and *Diphylla ecaudata* (GenBank Access Number: MK314979), and non-hematophagous bats *C. perspicillata* (GenBank Access Number: KY356757) and *S. lillium* (GenBank Access Number: KY356755) from Brazil. The remaining two sequences showed 91.72%–91.81% identity (query coverage of 99%–100%) to *Bartonella* spp. detected in *Cervus nippon* from Japan (GenBank Access Number: CP019781).

The five *rpoB* sequences obtained showed 85.66%–90.08% identity (query coverage of 99%–100%) to *Bartonella* spp. detected in a blood sample from *Eidolon helvum* bat (GenBank Access Number: KM215201) and to *Bartonella taylorii* detected in *Apodemus flavicollis* from Lithuania (GenBank Access Number: MH547315).

Finally, the three *ftsZ* sequences obtained showed 91.42% to 92.48% identity (query coverage of 100%) to *Bartonella* spp. detected in rodents (*Meriones libycus*) and bats (*Rhinolophus euryale*) from Georgia (GenBank Access Numbers: KT327035 e KX300105) and in the bat *E. helvum* from Tanzania (GenBank Access Number: KJ999693).

### 3.3. Genotype Analysis

**Figure 1:**
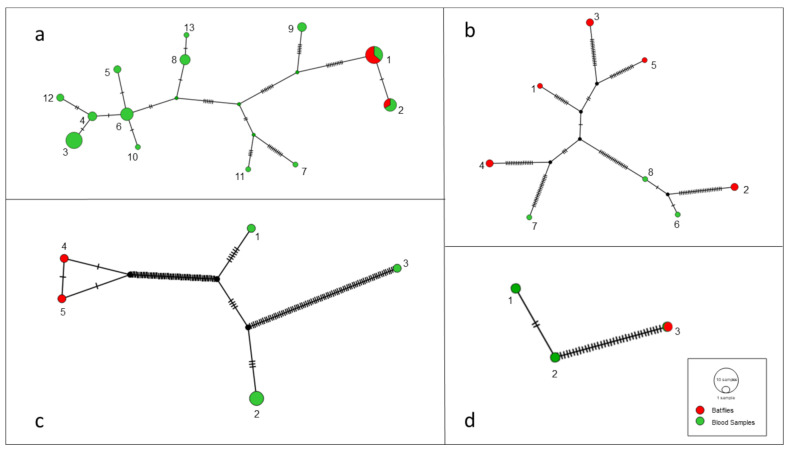
Genotype network with *gltA* (**a**), *nuoG* (**b**), *rpoB* (**c**) and *ftsZ* (**d**) sequences obtained from bat blood and ectoparasite samples obtained in the present study.

The results found for the 51 *gltA*-cloned sequences, which were obtained from 17 *Bartonella*-positive samples, showed the presence of at least two different genotypes among the three cloned sequences from each bat fly or bat blood sample (Table 1). However, the number of variable sites found among the genotypes was high (at least 20 variable sites) in only 6/17 cloned samples (blood samples), while the others (8 from blood samples and 3 from bat flies) showed 1 to 9 variable sites. Thus, even with more than one genotype per sample, the sequences obtained from the blood of animals #05, #20, #24, #32, #81 and #114 (all from the same bat species, namely *P. lineatus*) showed a greater number of nucleotide differences among them. When all the 51 cloned *gltA* sequences obtained were analyzed together, 13 different genotypes were observed (Figure 1a). In the genotype network generated for this gene fragment, a higher diversity was observed among the sequences obtained from bat blood samples when compared to those obtained from bat flies. Among the nine sequences obtained from three bat flies (*T. dugesii complex*), two different genotypes (1 and 2) were found. Only one ectoparasite had the cloned sequences separate in the two genotypes. These two abovementioned genotypes were also represented by sequences obtained from bat blood samples. The genotype 1 was found in blood samples from bats #05 with two clones, #20 with one clone, and #32 with one clone, as well as in bat flies #79 and #83, both with the three clones. The genotype 2 was represented by sequences found in blood samples from bats #18 with three clones, #114 with one clone, and in bat fly #30 with one clone.

In addition, polymorphism divergence analysis was performed between the group of sequences obtained from ectoparasites and bat blood samples and no significant difference between the two groups was found (Table 2).

The genotype diversity analysis of the *nuoG* (Figure 1b), *rpoB* (Figure 1c) and *ftsZ* (Figure 1c) sequences demonstrated the presence of eight, five and three distinct genotypes, respectively, among 11 sequences for the *nuoG* gene, seven for the *rpoB* gene and three for the *ftsZ* gene. In the genotype network generated for these gene fragments, the sequences obtained from bat flies were grouped in different groups from those obtained from bat blood samples as previously observed among *gltA* sequences.

All the results from the genotype analyses are shown in Table 3.

### 3.4. Phylogenetic Analysis

**Figure 2:**
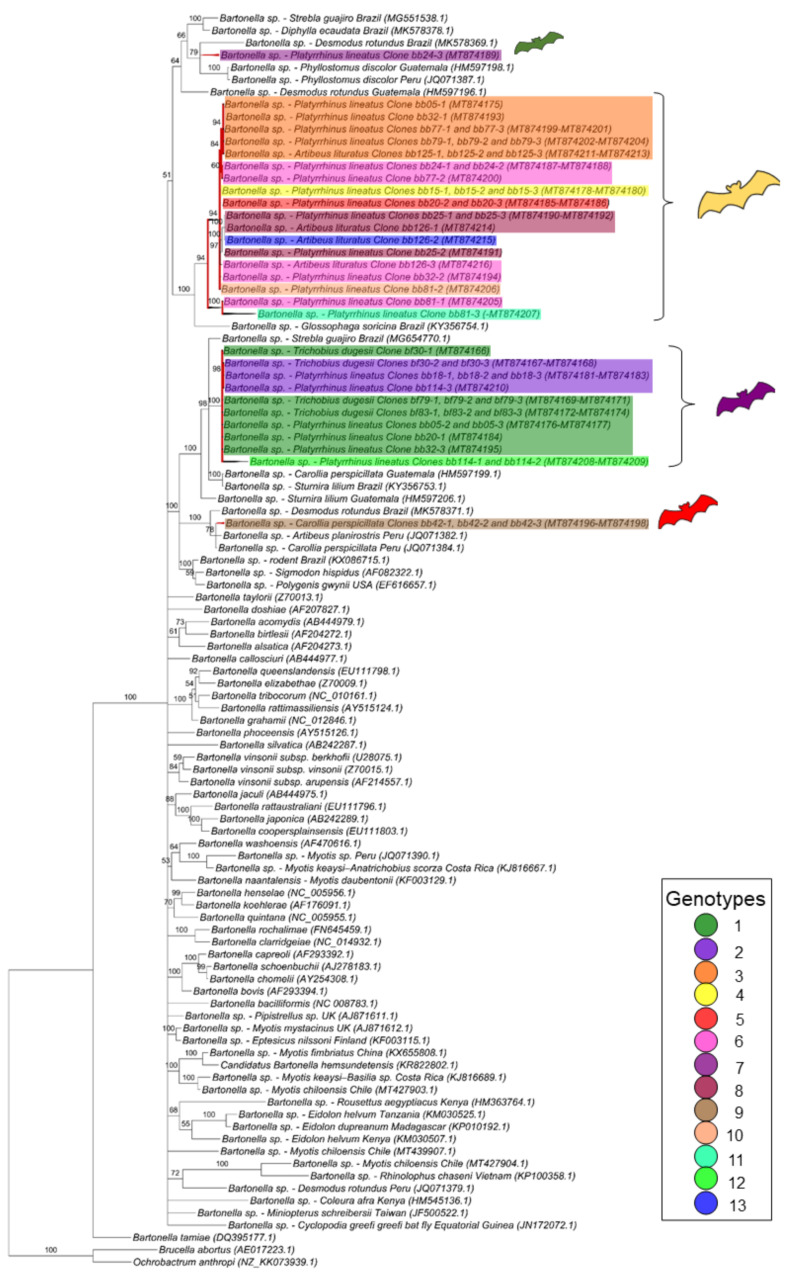
Phylogenetic tree based on an alignment of 790 bp-length of *Bartonella* spp. *gltA* sequences, using the Bayesian Inference method and TVM+I+G as the evolutionary model. Numbers at nodes correspond to the posterior probability value support. Each sequence-clone or group of sequences-clone are highlighted according to their respective genotype. The colored bats are positioned according to the groups found in the genealogy network. Each sample is designated with the type of sample (bb = bat blood and bf = bat fly) followed by the referred animal and clone numbers.

For the *gltA* gene fragment (790 bp), the *Bartonella* sequences were allocated in four groups into two large clades (Figure 2). The first one, containing only *Bartonella* sequences obtained from bats and including nine genotypes found in the present work, comprised two paraphyletic groups: one containing eight genotypes (3, 4, 5, 6, 8, 10, 11 and 13) closely related to each other, whereas the other one contained the genotype 7 and represented by only one clone from the bat blood sample #24. The other sequences obtained from this same bat specimen (blood sample #24) were positioned in the first paraphyletic group and represented by genotype 6, thus showing that the same bat blood sample contained more than one *Bartonella* genotype.

**Figure 3:**
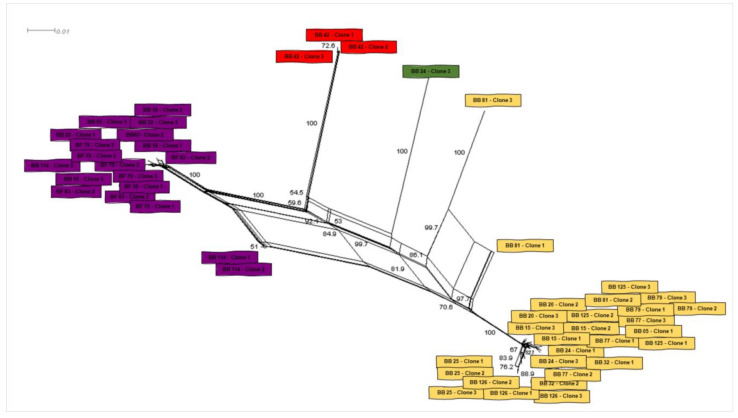
Genealogy network analysis of *Bartonella* spp. *gltA* sequences-clones obtained from bat blood and flies sampled in the present study. The analysis was performed with Splitstree software using the parameters “Neighbor-Net” with bootstrap of 1000 replicates.

The second large clade showed that the four remaining genotypes (1, 2, 9 and 12) were phylogenetically related to *Bartonella* sequences previously identified in rodents and other bats. In this clade, one genotype (9), which was represented by three clones from the same animal (bat blood #42), was positioned together with sequences previously detected in other bats from Peru and Brazil. The other three genotypes (1, 2 and 12), which comprised all the *Bartonella gltA* sequences obtained from ectoparasites as well as five sequences detected in bat blood samples, showed to be closely related to a *Bartonella* sequence obtained from *Strebla guajiro* in Brazil and other bat species from Brazil and Guatemala. Moreover, the 51 *gltA*-cloned sequences were submitted to a genealogy network analysis (Figure 3) and were distributed following the same pattern observed in the phylogenetic tree, where the bat figures represent each group found by different colors.

**Figure 4:**
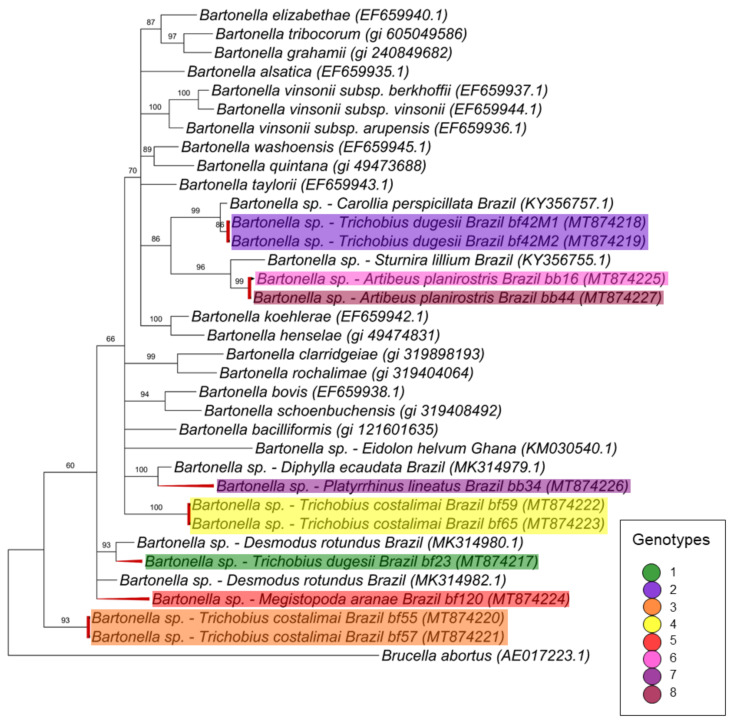
Phylogenetic tree based on an alignment of 368 bp-length of *Bartonella* spp. *nuoG* sequences, using the Bayesian Inference method and TIM2ef+I+G as the evolutionary model. Numbers at nodes correspond to the posterior probability value support. Each sequence is highlighted according to its respective genotype. Each sample is designated with the type of sample (bb = bat blood and bf = bat fly) followed by the referred animal number.

For the *nuoG* gene (368 pb) (Figure 4), seven different clusters were observed: the first and second ones are monophyletic and comprise three genotypes, genotype 2 with two sequences obtained from *T. dugesii complex* (#42M1 and #42M2) collected from the same animal (#42), and the other two genotypes (6 and 8) with sequences obtained from blood samples from the same animal species (*A. planirostris*) but different individuals (#16 and #44) that were positioned as monophyletic groups with 86% of posterior probability. Two *nuoG Bartonella* sequences from *C. perspicillata* and *S. lilium* previously sampled in Brazil were also positioned in the same branch. Two other genotypes (4 and 7), which comprised a sequence detected in *P. lineatus* (#34) and two sequences from *T. costalimai* (#59 and #56F1), were positioned as polytomic branches related to *Bartonella clarridgeiae*, *B. rochalimae*, *B. bacilliformis*, ruminant-related *Bartonella* and sequences previously detected in bats from Brazil and Ghana. The genotypes 1 and 5 were positioned as basal groups and comprised one sequence from *T. dugesii complex* (#23) and one from *M. aranea* (#120) that appeared to be closely related to *Bartonella* identified in vampire bats from Brazil. The last genotype comprising two sequences from *T. costalimai* flies (#55 and #57M) were positioned as one group basal from the others.

**Figure 5:**
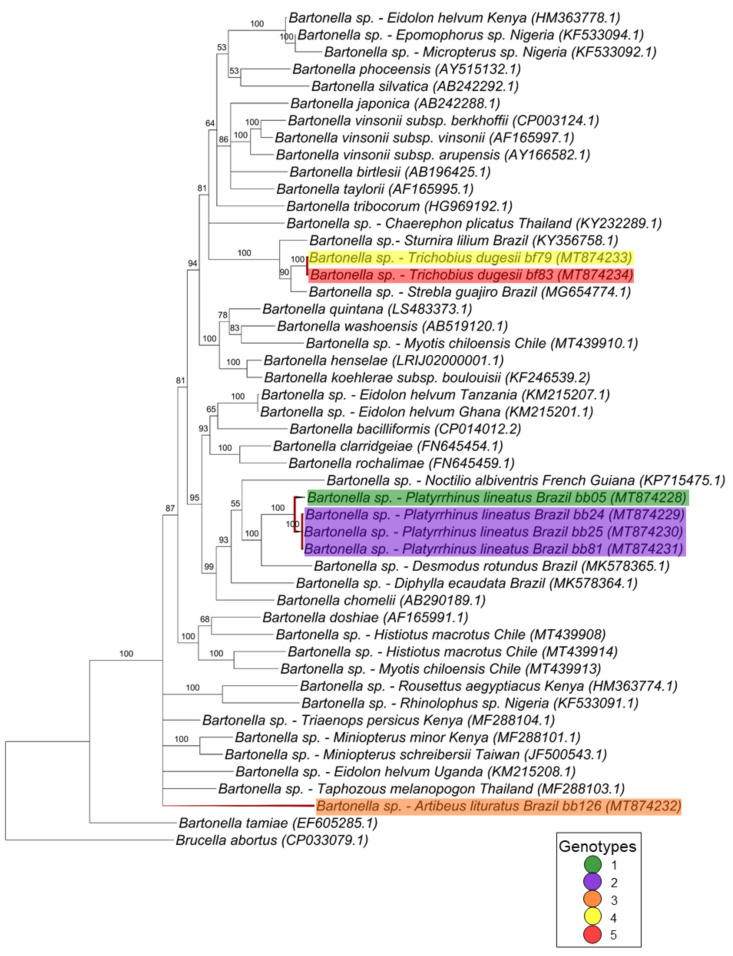
Phylogenetic tree based on an alignment of 757 bp-length of *Bartonella* spp. *rpoB* sequences, using the Bayesian Inference method and TrN+I+G as the evolutionary model. Numbers at nodes correspond to the posterior probability value support. Each sequence is highlighted according to its respective genotype. Each sample is designated with the type of sample (bb = bat blood and bf = bat fly) followed by the referred animal number.

In the phylogenetic analysis based on the *rpoB* gene (757 pb), the sequences were positioned in three separate clades (Figure 5). The first clade comprises two genotypes (4 and 5) with one sequence each and obtained from *T. dugesii* flies (#79 and #83) that were closely related to *Bartonella* genotypes previously detected in a bat and in a bat fly from Brazil, supported by 100% of posterior probability. The second clade, which was supported by 100% of posterior probability, was composed by four sequences detected in blood samples from *P. lineatus* (#05, #24, #25 and #81) and represented by two different genotypes (1 and 2). The most closely related sequences in this clade were represented by sequences obtained from bats from French Guiana and Brazil and *Bartonella chomellii*. The last sequence represented by genotype 3 and obtained from the blood sample of an *A. lituratus* individual appeared in a polytomic branch with *Bartonella* spp. detected in other bat species from Africa and Asia.

**Figure 6:**
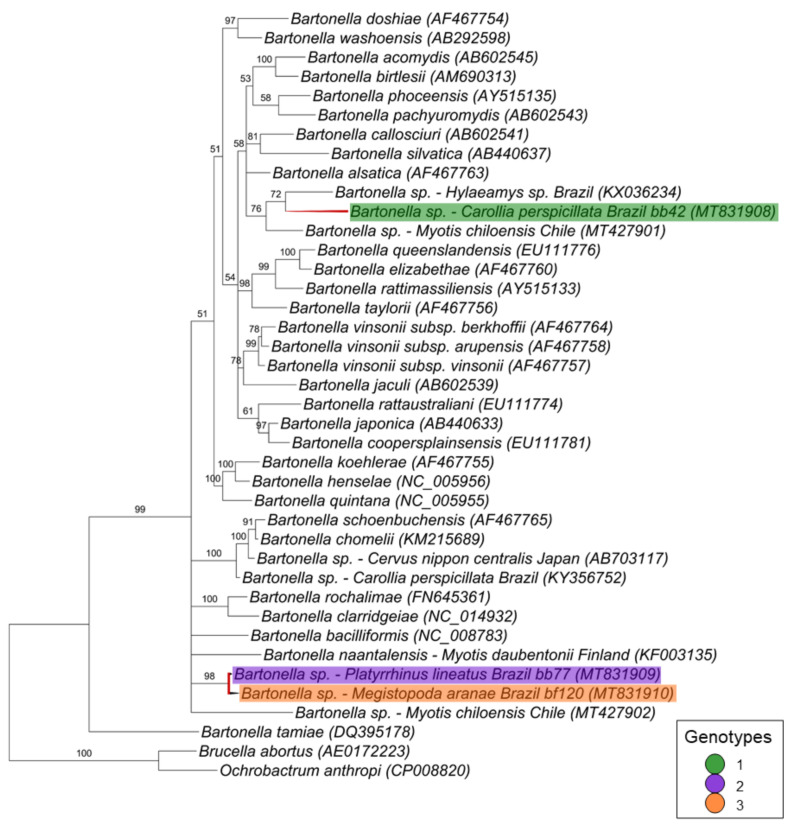
Phylogenetic tree based on an alignment of 521 bp fragment of *Bartonella* spp. *ftsZ* sequences, using the Bayesian Inference method and the TIM1+I+G as evolutionary model. Numbers at nodes correspond to the posterior probability value support. Each sequence is highlighted according to its respective genotype. Each sample is designated with the type of sample (bb = bat blood and bf = bat fly) followed by the referred animal number.

Finally, for the *ftsZ* gene fragment (521 bp), the three sequences corresponding to one genotype each were positioned in two different branches (Figure 6). The first branch comprised the genotype 1 (represented by a sequence detected in a bat blood sample [*C. perspicillata* #42]) that clustered together with *Bartonella* spp. detected in a rodent from Brazil and in a bat from Chile with 76% of posterior probability. The other two sequences, which were represented by genotypes 2 (represented by a sequence detected in a blood sample from *P. lineatus* #77) and 3 (represented by a sequence detected in *M. aranea* sample #120), clustered together in a polytomic branch—supported by 96% of posterior probability—with *Bartonella bacilliformis, Bartonella naantalensis* and *Bartonella* spp. detected in *Myotis chiloensis* from Chile. 

## 4. Discussion

Genotypic diversity of *Bartonella* spp. in bats and associated ectoparasites have been widely observed and reported [6,34,35,36,37,38,39]. In Brazil, previous studies showed the genetic diversity of bartonellae among non-hematophagous bats [12], and Streblidae flies [41], by phylogenetic analyses based on the *gltA* gene as well among vampire bats by phylogenetic and genotypic analysis based on *gltA* and *rpoB* genes [40]. However, while the majority of studies in the literature has focused on the analysis of genetic diversity of bartonellae in bats and their associated ectoparasites by targeting different *Bartonella* housekeping genes, as previously proposed [42], the present work aimed to investigate such diversity within both vertebrate and invertebrate hosts at an intra-host perspective. For such qn investigation, the *gltA* gene was chosen as it is the most frequently used molecular marker and is considered a good tool to evaluate the genotype diversity [42,73]. Even more, the number of *Bartonella gltA* sequences in GenBank is high and most frequently updated. La Scola et al., (2003) [73] also indicated the *gltA* together with the *rpoB* gene as suitable molecular markers for *Bartonella* differentiation and characterization. So, in paralell, we also assessed the genetic diversity by targeting three additional molecular markers, namely *rpoB*, *ftsZ* and *nuoG*.

Interestingly, while 18 bat blood samples showed to be positive for *Bartonella* spp., all spleen samples were negative. Despite the fact that spleen samples are considered a pertinent option for *Bartonella* detection, the blood is the most suitable choice sample for diagnosis of *Bartonella* spp. due to the hemotropism of this group of bacteria. Nevertheless, it is important to keep in mind that even blood samples can show false-negative results because of the cyclic bacteremia of bartonellae [74]. 

Considering that Nycteribiidae and Streblidae flies have been suggested as putative vectors for *Bartonella* among bats [75,76,77], the assessment of the genetic diversity of bartonellae among vertebrate hosts and associated ectoparasites as well as the possible exchange of *Bartonella-*genotypes between bats and related ectoparasites [75,78] has been increasingly addressed. In addition, McKee et al., (2016) [69], when evaluating the host-parasite interaction of *Bartonella* in bats, pointed out the existence of frequent and bidirectional exchange of this group of bacteria between bats and associated ectoparasites, highlighting the influence of arthropod vectors on the pattern of genetic variants of *Bartonella* spp. in vertebrate hosts. Herein, we observed such matched relationship only when we considered the genotypic analysis based on the *gltA* gene. In this sense, we found two *Bartonella* genotypes (1 and 2) that comprised sequences obtained from both the vertebrate host and ectoparasites.

However, when analyzing the other targeted gene fragments, the detected genotypes showed to be exclusive only to the vertebrate host or only to the sampled flies. In the phylogenetic analysis based on the *rpoB* and *nuoG* genes, we also observed that sequences obtained from ectoparasites samples clustered together among themselves and in different branches from those obtained from blood samples collected from their respective bat hosts. Even though one sequence from a bat blood sample (*P. lineatus* #77) and one sequence from *M. aranea* (#120) clustered together in the *ftsZ* gene phylogeny, both represent one genotype each. In fact, all the three sequences obtained from this marker were separated in three different genotypes. These findings might be explained by the symbiotic character of some *Bartonella* spp. in arthropods that are not present in vertebrate hosts, as previously shown [38,79].

Interestingly, in the *nuoG* phylogenetic tree, four sequences obtained from bat flies were positioned as more ancient groups. Previously, McKee et al. (2020) [29] raised the hypothesis that *Bartonella* species evolved from symbionts found in blood-feeding ectoparasites once these arthropods depend on symbionts for additional nutrients [80].

Despite the high genetic diversity of *Bartonella* spp. previously reported in bat ectoparasites [35], the present work found a lower genetic diversity among the *gltA* sequences obtained from flies when compared to those found in blood samples from bats. Only one bat fly sample showed more than one *Bartonella gltA* genotype among the three clones obtained from the same sample, while eight bat blood samples showed two or three different genotypes. In addition, *Bartonella* sequences detected in bat blood samples showed a higher number of variable sites, but without significant statistical difference when compared to sequences obtained from bat flies. Sandór et al. (2018) [81] proposed that a lower genetic diversity of *Bartonella* in bat flies could be related to a high exchange of hosts, since they would not be able to maintain a diversity of genotypes, despite having more access to it. On the other hand, flies with a lower host exchange rate would present a greater capacity to maintain the acquired genotypes since they would maintain them for a longer time. Conversely, some authors pointed out the possibility that a greater diversity of bartonellae in bat flies would be the opposite and related to the frequent change of hosts [38], as well as their lack of specificity [79], since this kind of behavior would allow the infection with different genotypes. Finally, we cannot rule out the possibility of greater *Bartonella* diversity among bats than in flies due to the number of samples analyzed, i.e., *gltA* amplicons for cloning were obtained from only three flies whereas 14 amplicons were obtained from bat blood samples. In addition, Becker et al. (2018) [39] suggested that the different levels of bartonellae genetic diversity between bats and their associated ectoparasites may also be related to the dispersion of this group of bacteria by other arthropod vectors or other bat species involved in the dynamic transmission cycles. However, as pointed out by McKee et al. (2020) [29], while strict ectoparasites vectors may maintain enzootic *Bartonella* infections, generalist vectors can carry the bacteria to other phylogenetically distant hosts. Moreover, the same authors incriminated bats as the likely key for *Bartonella* diversification and spreading. 

Mckee et al. (2019; 2020) [29,33] has shown that bat-associated *Bartonella* genotypes were scattered positioned in the phylogenetic trees versus those described in other mammal orders. Even though the *Bartonella* phylogeny is congruent with the host phylogeny, bat-related *Bartonella* clusters appeared to be polyphyletic, which is consistent to the topology observed in the phylogenetic analyses from the present work and from previous studies performed by our research group involving non-hematophagous bats [12], vampire bats [40], and Streblidae bat flies [41]. Herein, the sequences obtained from ectoparasites and bat samples appeared as branches distributed throughout the tree, showing that bartonellae detected in both bats and flies are very diverse. Even though *Bartonella* DNA detected in ectoparasites can be retrieved from the host as a product of blood meal, the cloned sequences obtained from the ectoparasite #79 clustered in a different branch from that where vertebrate host-associated sequences were positioned (bat blood sample #79) and therefore showed to be different from each other. Recently, Braga et al. (2020) [82] detected *Bartonella* DNA in 3 out 15 *Trichobius* spp. bat flies sampled in northeastern Brazil, despite the fact that all 29 blood samples from the associated bat hosts were negative. The *gltA* and *rpoB Bartonella* genotypes detected in that study were closely related to genotypes previously detected in bats from Brazil and Costa Rica [82]. It is important to highlight that the detection of *Bartonella* DNA in ectoparasites does not prove the vectorial competence and capacity of a certain vector for this group of bacteria. Also, we could observe that the found *Bartonella* sequences usually clustered together or were closely related to sequences previously obtained from bats and ectoparasites from Peru, Guatemala, French Guiana and Brazil. This clustering pattern was previously observed when Stuckey et al. (2017) [83] analyzed *Bartonella* sequences from bats from Mexico, which were positioned along with sequences obtained from previous works performed in Latin America. Similarly, Muller et al. (2020) [84] also showed a high diversity of *Bartonella* genotypes in bats from Chile, which also clustered with bartonellae detected in bats from Central and South America. Interestingly, some genotypes clustered with sequences obtained from bats from Asia, Africa and Europe. Herein, one sequence (from a bat *P. lineatus* blood sample #77) was positioned in the same branch with a rodent-related *Bartonella* sequence in the *ftsZ* phylogenic analysis. This phylogenetic relatedness between *Bartonella* sequences from bats and rodents has already been observed by our research group [12,41] and recently by Muller et al. 2020 [82]. 

Although *Bartonella* has already been detected in Macronyssidae mites from rodents [85], few works have reported the occurrence of bartonellae in bat-associated mites. Here, we found positivity to *Bartonella* spp. in five out of 21 pooled samples from Macronyssidae mites (*Steatonyssus* spp.) and none of the 46 pooled Spinturnicidae mites. Reeves et al. (2006) [86] reported positivity for a *Bartonella* spp. showing 96% identicalness to *B. grahamii* in a *Steatonyssus* spp. mite collected from a bat. In Hungary, Hornok et al. (2012) [87] detected *Bartonella* spp. in 25% (1/4) Macronyssidae (*Steatonyssus ocidentalis*) and 100% (3/3) Spinturnicidae (*Spinturnix myoti*) mite pooled samples collected from *Myotis myotis* specimens. Recently, 28% of the *S. myoti* (Spinturnicidae) mite pooled samples collected from bats from Poland were positive to *Bartonella* spp. [88]. Previously in Brazil, do Amaral et al. (2018) [41] did not find positivity among 200 Macronyssidae and Spinturnicidae collected from bats in the state of Rio de Janeiro. It is important to highlight that the pooled mite samples that were positive for *Bartonella* were collected in PCR negative animals and did not yield positive results in the additional conventional PCR assays. 

None of the *Ornithodoros* tick larvae samples were positive to *Bartonella* spp. in the present study. Similarly, Tahir et al. (2016) [89] found all 107 larvae of *Ornithodoros hasei* from *Noctilio albiventris* collected in French Guiana to be negative for *Bartonella* spp. On the other hand, Davoust et al. (2016) [90] found four out of 105 *O. hasei* ticks positive for *Bartonella* spp. The ticks were collected from 4 *N. albiventris* also in French Guiana Noteworthy, zoonotic-related *Bartonella* has already been detected in ticks associated to bats previously. Lofti et al. (2005) [91] detected *Bartonella henselae* 16S-23S ITS in a single *Carios kelleyi* nymph sampled in residential and community buildings in Iowa, and Leulmi et al. (2016) [92] detected *Bartonella tamiae* in 12/19 *Ixodes vespertilionis* ticks collected from bats in Algeria. Even though *Bartonella* spp. has been detected in several tick species [93,94], there is no strong evidence that ticks can be competent vectors for *Bartonella* spp. [95,96]. So far, it has been shown that *Ixodes ricinus* tick may act as competent vectors for *B. henselae* in laboratory [97].

## 5. Conclusions

We showed the genetic diversity of *Bartonella* genotypes in bat blood and ectoparasite samples at both intra- and inter-hosts levels. Based on the *gltA* molecular marker, we found a high genotypic diversity in both bat and fly hosts, which may lead to a complex host-pathogen interaction and displays a new perspective on the role of these animals in the maintenance of *Bartonella* spp. in the environment. Herein, the results obtained for *rpoB*, *nuoG* and *ftsZ* markers highlight the consistency with previous works that showed the genetic diversity of *Bartonella* in bats and associated ectoparasites. Since bats represent a large and widespread group of mammals around the world and *Bartonella* appears to have high genotypic plasticity, these flying mammals have been pointed out as key hosts in the origin and spread of these important zoonotic bacteria.

## Figures and Tables

**Table 1:** Polymorphism found among the three *Bartonella gltA* cloned sequences obtained from each bat blood and fly sample.

Sample	(pb)	N	VS	h	Hd (mean ± SD)	π (mean ± SD)	K
Bat fly #30	716	3	1	2	0.667 ± 0.314	0.00093 ± 0.00044	0.66667
Bat fly #79	731	3	2	3	1.000 ± 0.272	0.00182 ± 0.00061	1.33333
Bat fly #83	734	3	2	2	0.667 ± 0.314	0.00182 ± 0.00086	1.33333
Blood #05	601	3	94	2	0.667 ± 0.314	0.10427 ± 0.04915	62.66667
Blood #15	734	3	1	2	0.667 ± 0.314	0.00091 ± 0.00043	0.66667
Blood #18	528	3	3	3	1.0000 ± 0.272	0.00379 ± 0.00133	2
Blood #20	650	3	102	2	0.667 ± 0.314	0.10462 ± 0.04932	68
Blood #24	662	3	87	2	0.667 ± 0.314	0.08761 ± 0.04130	58
Blood #25	739	3	4	3	1.0000 ± 0.272	0.00361 ± 0.00134	2.66667
Blood #32	549	3	90	3	1.0000 ± 0.272	0.10929 ± 0.04870	60
Blood #42	674	3	1	2	0.667 ± 0.314	0.00099 ± 0.00047	0.66667
Blood #77	568	3	2	2	0.667 ± 0314	0.00235 ± 0.00111	1.33333
Blood #79	734	3	1	2	0.667 ± 0.314	0.00091 ± 0.00043	0.66667
Blood #81	782	3	86	3	1.000 ± 0.272	0.07332 ± 0.02460	57.33333
Blood #114	377	3	25	2	0.667 ± 0.314	0.04421 ± 0.02084	16.66667
Blood #125	719	3	1	2	0.667 ± 0.314	0.00093 ± 0.00044	0.66667
Blood #126	674	3	9	3	1.000 ± 0.272	0.00890 ± 0.00339	6

N: number of sequences analyzed; VS: number of variable sites; h: number of genotypes; Hd: genotype diversity; π: nucleotide diversity; K: nucleotide difference number; SD: standard deviation.

**Table 2:** Polymorphism found within each sample population (ectoparasite and bat blood samples) for *gltA Bartonella* sequences.

Population Analyzed	N	PS	π	k
Ectoparasites	9	1	0	0.389
Blood Samples	42	39	0	12.668

N: number of sequences analyzed; PS: number of polymorphic sites; π: nucleotide diversity; K: nucleotide difference number.

**Table 3:** Polymorphism found among the sequences obtained from ectoparasite and bat blood samples for each gene analyzed in the present study.

Gene	N	(pb)	VS	h	Hd (mean ± SD)	π (mean ± SD)	K
*gltA*	51	154	39	13	0.887 ± 0.022	0.09499 ± 0.00536	14.53412
*nuoG*	11	283	72	8	0.945 ± 0.054	0.11147 ± 0.00709	31.54545
*rpoB*	7	518	115	5	0.857 ± 0.138	0.10195 ± 0.02417	53.80952
*ftsZ*	3	239	35	3	1 ± 0.272	0.09902 ± 0.04410	23.66667

N: number of sequences analyzed; VS: number of variable sites; h: number of genotypes; Hd: genotype diversity; π: nucleotide diversity; K: nucleotide difference number; SD: standard deviation.

## Supplementary Materials

The following are available online at https://www.mdpi.com/2076-2607/8/11/1822/s1, Table S1. Identification of non-hematophagous bat species and associated ectoparasites sampled in Campo Grande city, state of Mato Grosso do Sul, Brazil. Table S2. Results of quantification and positivity of bat blood and ectoparasite samples tested for *Bartonella* spp. by qPCR (*nuoG* gene) and conventional (c) PCR assays for the *gltA*, *rpoB*, *ftsZ* and *nuoG* genes. The sequenced samples are highlighted in dark yellow. The sequenced samples are highlighted in dark yellow, Table S3. Results of nBLAST analysis with correspondent GenBank acession number found for each sequenced clone obtained from bat blood and fly samples with the correspondent genotype for each sample.
