# Supplementary material for: Intra- and Inter-Host Assessment of Bartonella Diversity with Focus on Non-Hematophagous Bats and Associated Ectoparasites from Brazil
Source: Microorganisms. 2020 Nov 19;8(11):1822. doi: 10.3390/microorganisms8111822 (PMC7699196; doi:10.3390/microorganisms8111822)
Supplement: Supplementary file 1 [file microorganisms-08-01822-s001.pdf]

Table S1. Identification of non-hematophagous bat species and associated ectoparasites sampled in Campo Grande city, state of Mato Grosso do Sul, Brazil.

**Animal**	**Bat species**	**Locality**	**Ectoparasites**
MCG 1	*Artibeus lituratus*	CEA Polonês	*Periglischrus iheringi*
MCG 2	*Artibeus lituratus*	CEA Polonês	*Paratrichobius longicrus*
			Periglischrus iheringi
MCG 3	*Artibeus planirostris*	CEA Polonês	*w/o*
MCG 4	*Myotis nigricans*	CEA Polonês	*Steatonyssus* spp.
MCG 5	*Platyrrhinus lineatus*	CEA Polonês	*Periglischrus iheringi*
			Steatonyssus spp.
MCG 6	*Artibeus planirostris*	CEA Polonês	*w/o*
MCG 7	*Artibeus planirostris*	CEA Polonês	(2) *Megistopoda aranea*
MCG 8	*Artibeus lituratus*	CEA Polonês	*Megistopoda aranea*
			Periglischrus iheringi
MCG 9	*Glossophaga soricina*	CEA Polonês	*Trichobius parasiticus (complex)*
MCG 10	*Artibeus lituratus*	CEA Polonês	*Megistopoda aranea*
MCG 11	*Artibeus planirostris*	CEA Polonês	*Megistopoda aranea*
			Periglischrus iheringi
MCG 12	*Artibeus planirostris*	CEA Polonês	*Megistopoda aranea*
MCG 13	*Artibeus planirostris*	CEA Polonês	*w/o*
MCG 14	*Artibeus planirostris*	CEA Polonês	*Periglischrus iheringi*
MCG 15	*Platyrrhinus lineatus*	CEA Polonês	*w/o*
MCG 16	*Artibeus planirostris*	CEA Polonês	*Megistopoda aranea*
MCG 17	*Artibeus planirostris*	CEA Polonês	*Megistopoda aranea*
MCG 18	*Platyrrhinus lineatus*	CEA Polonês	*Periglischrus iheringi*
MCG 19	*Artibeus planirostris*	CEA Polonês	(2) *Megistopoda aranea*
MCG 20	*Platyrrhinus lineatus*	CEA Polonês	*Trichobius dugesii (complex)*
MCG 21	*Platyrrhinus lineatus*	CEA Polonês	*Periglischrus iheringi*
MCG 22	*Platyrrhinus lineatus*	CEA Polonês	*w/o*
MCG 23	*Eptesicus furinalis*	CEA Polonês	*Trichobius dugesii (complex)*
			Steatonyssus spp.
MCG 24	*Platyrrhinus lineatus*	CEA Polonês	*w/o*
MCG 25	*Platyrrhinus lineatus*	CEA Polonês	*Trichobius dugesii (complex)*

			Periglischrus iheringi
MCG 26	*Artibeus planirostris*	CEA Polonês	*Periglischrus iheringi*
MCG 27	*Artibeus planirostris*	CEA Polonês	*Megistopoda aranea*
			Periglischrus iheringi
MCG 28	*Artibeus lituratus*	CEA Polonês	*Periglischrus iheringi*
MCG 29	*Artibeus planirostris*	CEA Polonês	*w/o*
MCG 30	*Platyrrhinus lineatus*	CEA Polonês	*Trichobius dugesii (complex)*
MCG 31	*Artibeus lituratus*	CEA Polonês	*w/o*
MCG 32	*Platyrrhinus lineatus*	CEA Polonês	*Periglischrus iheringi*
MCG 33	*Artibeus planirostris*	CEA Polonês	*Periglischrus iheringi*
MCG 34	*Platyrrhinus lineatus*	CEA Polonês	*Trichobius parasiticus (complex)*
			Periglischrus iheringi
MCG 35	*Artibeus planirostris*	CEA Polonês	*Periglischrus iheringi*
MCG 36	*Artibeus planirostris*	CEA FLorestinha	*w/o*
MCG 37	*Carollia perspicillata*	CEA Florestinha	*Trichobius joblingi*
MCG 38	*Artibeus planirostris*	CEA Florestinha	*Megistopoda aranea*
MCG 39	*Artibeus planirostris*	CEA Florestinha	*Megistopoda aranea*
MCG 40	*Artibeus planirostris*	CEA Florestinha	*Megistopoda aranea*
MCG 41	*Platyrrhinus lineatus*	CEA Florestinha	*Periglischrus iheringi*
MCG 42	*Carollia perspicillata*	CEA Florestinha	(2) *Trichobius dugesii (complex)*
MCG 43	*Artibeus planirostris*	CEA Florestinha	*Megistopoda aranea*
MCG 44	*Artibeus planirostris*	CEA Polonês	*Megistopoda aranea*
MCG 45	*Myotis nigricans*	CEA Polonês	*w/o*
MCG 46	*Artibeus lituratus*	CEA Polonês	*w/o*
MCG 47	*Platyrrhinus lineatus*	CEA Polonês	*w/o*
MCG 48	*Artibeus planirostris*	CEA Polonês	*w/o*
MCG 49	*Artibeus lituratus*	CEA Polonês	*w/o*
MCG 50	*Artibeus lituratus*	CEA Polonês	*w/o*
MCG 51	*Artibeus planirostris*	CEA Polonês	*w/o*
MCG 52	*Molossus molossus*	CEA Polonês	*w/o*
MCG 53	*Artibeus lituratus*	CEA Polonês	*w/o*
MCG 54	*Molossus molossus*	CEA FLorestinha	*w/o*
MCG 55	*Phyllostomus discolor*	CEA Florestinha	*Trichobius costalimai*

MCG 56	*Phyllostomus discolor*	CEA Florestinha	*Strebla hertigi*
			(4) *Trichobius costalimai*
			Periglischrus torrealbai
			Periglischrus acutisternus
			Steatonyssus spp.
			(6) *Ornithodoros hasei*
MCG 57	*Phyllostomus discolor*	CEA Florestinha	(5) *Trichobius costalimai*
			Periglischrus torrealbai
			(2) *Ornithodoros hasei*
MCG 58	*Artibeus lituratus*	CEA Florestinha	*Periglischrus iheringi*
MCG 59	*Phyllostomus discolor*	CEA Florestinha	*Trichobius costalimai*
			Periglischrus spp.
MCG 60	*Molossops temminckii*	CEA Florestinha	*w/o*
MCG 61	*Phyllostomus discolor*	CEA Florestinha	*Periglischrus torrealbai*
			Ornithodoros hasei
MCG 62	*Phyllostomus discolor*	CEA Florestinha	*Trichobius costalimai*
			(2) *Periglischrus* spp.
			(2) *Ornithodoros hasei*
MCG 63	*Phyllostomus discolor*	CEA Florestinha	(2) *Trichobius costalimai*
			(7) *Ornithodoros hasei*
			Steatonyssus spp.
MCG 64	*Phyllostomus discolor*	CEA Florestinha	(3) *Trichobius costalimai*
			Steatonyssus spp.
MCG 65	*Phyllostomus discolor*	CEA Florestinha	(4) *Trichobius costalimai*
			Periglischrus acutisternus
MCG 66	*Phyllostomus discolor*	CEA Florestinha	(2) *Trichobius costalimai*
			Periglischrus acutisternus
MCG 67	*Phyllostomus discolor*	CEA Florestinha	*Periglischrus torrealbai*
MCG 68	*Phyllostomus discolor*	CEA Florestinha	(3) *Trichobius costalimai*
			P. torrealbai
MCG 69	*Carollia perspicillata*	CEA Florestinha	*Trichobius joblingi*
MCG 70	*Phyllostomus discolor*	CEA Florestinha	(2) *Strebla hertigi*
			(3) *Periglischrus* spp.

MCG 71	*Phyllostomus discolor*	CEA Florestinha	*Periglischrus* spp.
			Steatonyssus spp.
			Trichobius costalimai
MCG 72	*Phyllostomus discolor*	CEA Florestinha	*Trichobius costalimai*
			Periglischrus acutisternus
			Ornithodoros hasei
MCG 73	*Molossus molossus*	CEA Florestinha	*w/o*
MCG 74	*Eptesicus furinalis*	CEA Polonês	*Steatonyssus* spp.
MCG 75	*Eptesicus furinalis*	CEA Polonês	*Steatonyssus* spp.
MCG 76	*Artibeus lituratus*	CEA Polonês	*w/o*
MCG 77	*Platyrrhinus lineatus*	CEA Polonês	*w/o*
MCG 78	*Artibeus lituratus*	CEA Polonês	*Periglischrus iheringi*
MCG 79	*Platyrrhinus lineatus*	CEA Polonês	*Trichobius dugesii (complex)*
MCG 80	*Platyrrhinus lineatus*	CEA Polonês	*w/o*
MCG 81	*Platyrrhinus lineatus*	CEA Polonês	*w/o*
MCG 82	*Eptesicus furinalis*	CEA Polonês	*Steatonyssus* spp.
MCG 83	*Platyrrhinus lineatus*	CEA Polonês	*Trichobius dugesii (complex)*
			Periglischrus iheringi
MCG 84	*Artibeus lituratus*	CEA Polonês	*Periglischrus iheringi*
MCG 85	*Artibeus lituratus*	CEA Polonês	*w/o*
MCG 86	*Platyrrhinus lineatus*	CEA Polonês	*w/o*
MCG 87	*Artibeus lituratus*	CEA Polonês	*Periglischrus iheringi*
MCG 88	*Platyrrhinus lineatus*	CEA Polonês	*w/o*
MCG 89	*Platyrrhinus lineatus*	CEA Polonês	*Trichobius dugesii (complex)*
MCG 90	*Artibeus lituratus*	CEA Polonês	*Periglischrus iheringi*
			Steatonyssus spp.
MCG 91	*Eumops perotis*	CEA Florestinha	*Steatonyssus* spp.
MCG 92	*Eumops perotis*	CEA Florestinha	*Steatonyssus* spp.
MCG 93	*Eumops perotis*	CEA Florestinha	*Steatonyssus* spp.
MCG 94	*Molossus molossus*	CEA Florestinha	*Steatonyssus* spp.
MCG 95	*Eumops perotis*	CEA Florestinha	*Steatonyssus* spp.
MCG 96	*Artibeus lituratus*	CEA Polonês	*w/o*
MCG 97	*Artibeus lituratus*	CEA Polonês	*Periglischrus iheringi*

MCG 98	*Artibeus lituratus*	CEA Polonês	*w/o*
MCG 99	*Chiroderma villosum*	CEA Polonês	*w/o*
MCG 100	*Artibeus lituratus*	CEA Polonês	*w/o*
MCG 101	*Platyrrhinus lineatus*	CEA Polonês	*w/o*
MCG 102	*Artibeus lituratus*	CEA Polonês	*Periglischrus iheringi*
MCG 103	*Artibeus planirostris*	CEA Polonês	*w/o*
MCG 104	*Artibeus lituratus*	CEA Polonês	*Periglischrus iheringi*
MCG 105	*Artibeus lituratus*	CEA Polonês	*w/o*
MCG 106	*Eptesicus furinalis*	CEA Polonês	*Steatonyssus* spp.
MCG 107	*Artibeus planirostris*	CEA Polonês	*w/o*
MCG 108	*Artibeus planirostris*	CEA Polonês	*w/o*
MCG 109	*Artibeus planirostris*	CEA Polonês	*w/o*
MCG 110	*Artibeus planirostris*	CEA Polonês	*w/o*
MCG 111	*Carollia perspicillata*	CEA Polonês	*w/o*
MCG 112	*Artibeus planirostris*	CEA Polonês	*w/o*
MCG 113	*Artibeus planirostris*	CEA Polonês	*w/o*
MCG 114	*Platyrrhinus lineatus*	CEA Polonês	*w/o*
MCG 115	*Artibeus planirostris*	CEA Florestinha	*w/o*
MCG 116	*Artibeus lituratus*	CEA Florestinha	*w/o*
MCG 117	*Artibeus lituratus*	CEA Florestinha	*w/o*
MCG 118	*Artibeus lituratus*	CEA Florestinha	*w/o*
MCG119	*Artibeus lituratus*	CEA Florestinha	*w/o*
MCG 120	*Artibeus planirostris*	CEA Florestinha	*Megistopoda aranea*
MCG 121	*Artibeus lituratus*	CEA Florestinha	*w/o*
MCG 122	*Artibeus planirostris*	CEA Polonês	*Steatonyssus* spp.
MCG 123	*Eptesicus furinalis*	CEA Polonês	*Steatonyssus* spp.
MCG 124	*Eptesicus furinalis*	CEA Polonês	*Periglischrus* spp.
MCG 125	*Artibeus lituratus*	CEA Polonês	*w/o*
MCG 126	*Artibeus lituratus*	CEA Polonês	*Trichobius dugesii (complex)*
MCG 127	*Carollia perspicillata*	CEA Polonês	*w/o*
MCG 128	*Artibeus lituratus*	CEA Florestinha	*Periglischrus iheringi*
MCG 129	*Artibeus lituratus*	CEA Florestinha	*w/o*
MCG 130	*Artibeus lituratus*	CEA Florestinha	*w/o*

MCG 131	*Eptesicus furinalis*	CEA Polonês	*Steatonyssus* spp.
MCG 132	*Eptesicus furinalis*	CEA Polonês	*Steatonyssus* spp.
MCG 133	*Artibeus lituratus*	CEA Florestinha	*Periglischrus iheringi*
MCG 134	*Artibeus lituratus*	CEA Florestinha	*Periglischrus iheringi*
MCG 135	*Artibeus lituratus*	CEA Florestinha	*w/o*

w/o: ectoparasites were not found on bats at sampling.

Table S2. Results of quantification and positivity of bat blood and ectoparasite samples tested for *Bartonella* spp. by qPCR (*nuoG* gene) and conventional (c) PCR assays for the *gltA*, *rpoB*, *ftsZ* and *nuoG* genes. The sequenced samples are highlighted in dark yellow.

**Local**	**Species**	**Ectoparasite Host**	**Sample**	**qPCR quantification (*nuoG* copies/µL)**	**cPCR *gltA***	**cPCR *rpoB***	**cPCR *ftsZ***	**cPCR *nuoG***
CEA Polonês	*Platyrrhinus lineatus*		Blood #05	5.19 x 10^0 ± 5.00 x 10^-1	+	+	-	+NS
CEA Polonês	*Platyrrhinus lineatus*		Blood #15	3.02 x 10^0 ± 7.78 x 10^-1	+	-	-	+NS
CEA Polonês	*Artibeus planirostris*		Blood #16	1.86 x 10^0 ± 2.40 x 10^-1	+NS	+ F	+ F	+
CEA Polonês	*Platyrrhinus lineatus*		Blood #18	4.73 x 10^1 ± 1.08 x 10^0	+	+ F	+ F	+NS
CEA Polonês	*Platyrrhinus lineatus*		Blood #20	1.20 x 10^0 ± 1.55 x 10^-1	+	-	-	+NS
CEA Polonês	*Trichobius dugesii complex*	*Eptesicus furinalis*	Batfly #23	3.36 x 10^1 ± 6.28 x 10^0	-	+ F	+ F	+
CEA Polonês	*Platyrrhinus lineatus*		Blood #24	1.80 x 10^1 ± 4.13 x 10^0	+	+	-	+NS
CEA Polonês	*Platyrrhinus lineatus*		Blood #25	9.57 x 10^1 ± 1.78 x 10^1	+	+	+ F	+NS
CEA Polonês	*Trichobius dugesii complex*	*Platyrrhinus lineatus*	Batfly #30	3.28 x 10^2 ± 6.49 x 10^0	+	+ F	-	+NS
CEA Polonês	*Platyrrhinus lineatus*		Blood #32	1.40 x 10^0 ± 1.59 x 10^-1	+	-	-	+NS
CEA Polonês	*Platyrrhinus lineatus*		Blood #34	1.20 x 10^0 ± 6.35 x 10^-2	+ F	-	-	+
CEA Florestinha	*Carollia perspicillata*		Blood #42	2.91 x 10^3 ± 1.77 x 10^2	+	-	+	+NS
CEA Florestinha	*Trichobius dugesii complex*	*Carollia perspicillata*	Batfly #42 M1	1.87 x 10^0 ± 2.98 x 10^-1	-	-	-	+
CEA Florestinha	*Trichobius dugesii complex*	*Carollia perspicillata*	Batfly #42 M2	1.04 x 10^0 ± 2.36 x 10^-1	-	-	-	+
CEA Polonês	*Artibeus planirostris*		Blood #44	1.09 x 10^1 ± 9.88 x 10^-1	+ F	+ F	+ F	+
CEA Polonês	*Artibeus planirostris*		Blood #51	8.14 x 10^-1 ± 2.60 x 10^-2	+NS	-	+ F	+NS

CEA Florestinha	*Trichobius costalimai*	*Phyllostomus discolor*	Batfly #55	9.84 x 10^-1 ± 2.03 x 10^-1	-	-	-	+
CEA Florestinha	*Trichobius costalimai*	*Phyllostomus discolor*	Batfly #57 M	1.69 x 10^-1 ± 4.53 x 10^-2	+ F	-	+ F	+
CEA Florestinha	*Trichobius costalimai*	*Phyllostomus discolor*	Batfly #59	1.94 x 10^0 ± 2.39 x 10^-1	+ F	-	+ F	+
CEA Florestinha	*Trichobius costalimai*	*Phyllostomus discolor*	Batfly #65 F1	1.77 x 10^0 ± 2.85 x 10^-1	-	-	-	+
CEA Polonês	*Steatonyssus spp.*	*Eptesicus furinalis*	Bat mite #74	1.07 x 10^0 ± 2.31 x 10^-1	-	-	+ F	-
CEA Polonês	*Steatonyssus spp.*	*Eptesicus furinalis*	Bat mite #75	3.51 x 10^-1 ± 7.8 x 10^-2	-	-	+ F	-
CEA Polonês	*Platyrrhinus lineatus*		Blood #77	2.95 x 10^0 ± 3.98 x 10^-1	+	-	+	+NS
CEA Polonês	*Platyrrhinus lineatus*		Blood #79	3.65 x 10^-1 ± 8.27 x 10^-3	+	-	-	+NS
CEA Polonês	*Trichobius dugesii complex*	*Platyrrhinus lineatus*	Batfly #79	1.29 x 10^3 ± 2.24 x 10^2	+	+	-	+NS
CEA Polonês	*Platyrrhinus lineatus*		Blood #81	1.97 x 10^0 ± 3.03 x 10^-1	+	+	-	+NS
CEA Polonês	*Steatonyssus spp.*	*Eptesicus furinalis*	Bat mite #82	9.75 x 10^-2 ± 3.51 x 10^-3	-	-	+ F	-
CEA Polonês	*Trichobius dugesii complex*	*Platyrrhinus lineatus*	Batfly #83	6.38 x 10^2 ± 2.40 x 10^1	+	+	+ F	+NS
CEA Polonês	*Platyrrhinus lineatus*		Blood #114	9.38 x 10^1 ± 1.58 x 10^-1	+	+ F	+ F	+NS
CEA Florestinha	*Megistopoda aranea*	*Artibeus planirostris*	Batfly #120	9.20 x 10^-1 ± 2.23 x 10^-1	-	-	+	+
CEA Polonês	*Artibeus lituratus*		Blood #125	6.43 x 10^0 ± 1.36 x 10^0	+	-	-	+NS
CEA Polonês	*Artibeus lituratus*		Blood #126	6.34 x 10^0 ± 1.37 x 10^0	+	+	-	+NS
CEA Polonês	*Steatonyssus spp.*	*Eptesicus cf. furinalis*	Bat mite #132	4.27 x 10^0 ± 8.38 x 10^-1	-	-	+ F	-

+ = positive and sequenced; +NS = positive but not sequenced; +F = positive with a faint band in electrophoresis agarose-gel; - = negative.

Table S3. Results of nBLAST analysis with correspondent GenBank acession number for each sequenced clone obtained from bat blood and fly samples with the correspondent genotype for each sample.

Animal	Local	Specie	Sample	Clone 1	Clone 2	Clone 3	Query Coverage	Identity	Genotypes
MCG 05	CEA Polonês	*Platyrrhinus lineatus*	Blood	*Bartonella* spp. (MH234319.1)	*Bartonella* spp. (MH234380.1)	*Bartonella* spp. (MH234380.1)	98-100%	93-95%	1 and 3
MCG 15	CEA Polonês	*Platyrrhinus lineatus*	Blood	*Bartonella* spp. (KJ816676.1)	*Bartonella* spp. (KJ816676.1)	*Bartonella* spp. (KJ816676.1)	95-99%	92%	4
MCG 18	CEA Polonês	*Platyrrhinus lineatus*	Blood	*Bartonella* spp. (KJ816687.1)	*Bartonella* spp. (MH234380.1)	*Bartonella* spp. (MH234380.1)	95-97%	95%	2
MCG 20	CEA Polonês	*Platyrrhinus lineatus*	Blood	*Bartonella* spp. (MH234347.1)	*Bartonella* spp. (KJ816676.1)	*Bartonella* spp. (KJ816676.1)	96-98%	92-96%	1 and 5
MCG 24	CEA Polonês	*Platyrrhinus lineatus*	Blood	*Bartonella* spp. (KJ816676.1)	*Bartonella* spp. (KJ816676.1)	*Bartonella* spp. (MH234333.1)	89-97%	92-99%	6 and 7
MCG 25	CEA Polonês	*Platyrrhinus lineatus*	Blood	*Bartonella* spp. (KJ816676.1)	*Bartonella* spp. (KJ816676.1)	*Bartonella* spp. (KJ816676.1)	94-98%	92%	8
MCG 30	CEA Polonês	*Trichobius dugesii complex*	Bat fly 30	*Bartonella* vinsonii (LR134529.1)	*Bartonella* vinsonii (LR134529.1)	*Bartonella* vinsonii (LR134529.1)	100%	88%	1 and 2
MCG 32	CEA Polonês	*Platyrrhinus lineatus*	Blood	*Bartonella* spp. (MH234319.1)	*Bartonella* spp. (KJ816676.1)	*Bartonella* spp. (MH234380.1)	98-99%	92-95%	1, 3 and 6
MCG 42	CEA Florestinha	*Carollia perspicillata*	Blood	*Bartonella* spp. (MH234346.1)	*Bartonella* spp. (MH234346.1)	*Bartonella* spp. (MH234346.1)	90-91%	99-100%	9
MCG 77	CEA Polonês	*Platyrrhinus lineatus*	Blood	*Bartonella* spp. (MH234319.1)	*Bartonella* spp. (KJ816682.1)	*Bartonella* spp. (KJ816682.1)	99-100%	93%	3 and 6
MCG 79	CEA Polonês	*Platyrrhinus lineatus*	Blood	*Bartonella* spp. (KJ816676.1)	*Bartonella* spp. (KJ816676.1)	*Bartonella* spp. (KJ816676.1)	95-97%	92-93%	3
		Trichobius dugesii complex	Bat fly 79	*Bartonella* spp. (MH234380.1)	*Bartonella* spp. (MH234380.1)	*Bartonella* spp. (MH234380.1)	95-97%	95%	1
MCG 81	CEA Polonês	*Platyrrhinus lineatus*	Blood	*Bartonella* spp. (KJ816673.1)	*Bartonella* spp. (KJ816676.1)	*Bartonella* spp. (MH234322.1)	90-96%	91-96%	6, 10 and 11
MCG 83	CEA Polonês	*Trichobius dugesii complex*	Bat fly 83	*Bartonella* spp. (MH234380.1)	*Bartonella* spp. (MH234380.1)	*Bartonella* spp. (MH234380.1)	96-98%	95%	1

MCG 114	CEA Polonês	*Platyrrhinus lineatus*	Blood	*Bartonella* spp. (MH234380.1)	*Bartonella* spp. (MH234380.1)	*Bartonella* spp. (MH234380.1)	95-97%	92-96%	2 and 12
MCG 125	CEA Polonês	*Artibeus lituratus*	Blood	*Bartonella* spp. (KJ816682.1)	*Bartonella* spp. (KJ816676.1)	*Bartonella* spp. (KJ816676.1)	97-99%	92%	3
MCG 126	CEA Polonês	*Artibeus lituratus*	Blood	*Bartonella* spp. (KJ816676.1)	*Bartonella* spp. (KJ816682.1)	*Bartonella* spp. (KJ816682.1)	97-99%	92%	6, 8 and 13
